# The Norwegian version of the Norwich Patellar Instability score has good validity and moderate reproducibility

**DOI:** 10.1002/jeo2.70095

**Published:** 2025-01-10

**Authors:** T. Hysing‐Dahl, A. G. Faleide, L. H. Magnussen, E. Inderhaug

**Affiliations:** ^1^ Department of Surgery Haraldsplass Deaconess Hospital Bergen Norway; ^2^ Sports Traumatology and Arthroscopy Research Group (STAR Group), Department of Clinical Medicine University of Bergen Bergen Norway; ^3^ Western Norway University of Applied Sciences Bergen Norway; ^4^ Department of Orthopaedic Surgery Haukeland University Hospital Bergen Norway

**Keywords:** COSMIN, Norwich Patellar Instability score, patellar instability, reliability, validity

## Abstract

**Purpose:**

To translate and adapt the Norwich Patellar Instability (NPI) score into Norwegian, and second, to examine the psychometric properties of the Norwegian version (NPI‐No).

**Methods:**

NPI was translated according to international guidelines. A cohort of 107 patients surgically treated for recurrent patellofemoral instability completed NPI‐No, related questionnaires and functional tests prior to and six months post‐surgery. Validity (face, content and construct validity), internal consistency (Cronbach's alpha [*α*]), test–retest reliability (intraclass correlation coefficient [ICC]], measurement error (standard error of measurement [SEM] and smallest detectable change at individual [SDC_ind_] and group level [SDC_group_]) and construct validity (hypotheses testing; independent *t* tests, Pearson's *r*) were examined.

**Results:**

NPI‐No had good face and content validity. Internal consistency was satisfactory (*α* = 0.88), test–retest reliability was moderate ICC_2.1_ 0.65 (95% confidence interval = 0.47–0.77) and measurement error low (SEM = 7.8). SDC_ind_ was 21.7 points and SDC_group_ was 2.8. Seven of the 10 hypotheses about construct validity were confirmed. While there was no ceiling effect pre‐ or post‐operatively, a substantial floor effect (28%) was observed at the 6‐month follow‐up.

**Conclusion:**

The NPI‐No is valid for assessment of self‐perceived patellar instability before and after surgery in Norwegian patients. However, reproducibility was found to be only moderate. This study adds further knowledge about the measurement properties of the NPI.

**Level of Evidence:**

Level II.

**Abbreviations**
BPII: Banff Patellofemoral Instability Instrument 2.0
CI: confidence interval
CM: centimetre
COSMIN: COnsensus‐based Standards for the selection of health Measurement Instruments
ICC: intraclass correlation coefficient
IKDC: International Knee Documentation Committee Subjective Knee Form
KOOS: Knee injury and Osteoarthritis Outcome Score
LoA: limits of agreement
MPFL‐R: medial patellofemoral ligament reconstruction
Nm: Newton metre
NPI: Norwich Patellar Instability
PROM: Patient Reported Outcome Measure
PT: peak torque
QOL: quality‐of‐life
SDC: smallest detectable change
SEM: standard error of measurement
TSK: Tampa Scale of Kinesiophobia
YBT‐LQ: lower quarter Y‐balance test

## INTRODUCTION

Patellar instability is a complex and multifaceted condition that has garnered increasing attention over the past few years [6, 8, 16, 26, 29, 30, 31]. The main symptom of the disorder is instability and therefore the goal of treatment, operative and non‐operative, is to reduce instability symptoms and improve function, enabling normal everyday life functioning and sports participation [6].

To evaluate treatment, including success or failure of surgical interventions, and patients‘ progression during rehabilitation, valid and reliable measurement instruments such as patient‐reported outcome measures (PROMs) are needed [7]. PROMs with satisfactory measurement properties allow healthcare providers to effectively monitor patients' progress, thereby optimizing the delivered treatment. In addition to more precise evaluation of different surgical techniques and treatment approaches in research [7]. Currently, the Norwich Patellar Instability (NPI) score [27, 28, 29] and the Banff Patellofemoral Instability Instrument (BPII) 2.0 [10, 15] are the only diagnose‐specific PROMs for patients with patellar instability [11]. Only the NPI directly evaluates physical symptoms, by assessing the severity of patient‐perceived patellar instability during activity [29]. The NPI score has demonstrated adequate construct validity, high internal consistency and responsiveness [10, 27, 28, 29]. Furthermore, is it the only instrument that has been validated in a first‐time dislocation population [27].

There are, however, measurement properties that needs further evaluation, particularly information on test–retest reliability including the reporting of measurement error that will enhance interpretation of scores. Further, knowledge on cross‐cultural validity and hypothesis testing is sparse. Although a few translations have been made [1, 33, 36], there is currently no translated and validated Norwegian version available.

The present study aimed to translate and adapt the NPI into Norwegian, and second, to examine validity (content and construct validity) and reliability (consistency, test– retest reliability and measurement error).

## MATERIALS AND METHODS

The study was approved by the Regional Committee for Medical and Health Research Ethics ID: 2020/185067) and the NSD (Norwegian Centre for Research Data) Data Protection Official for Research (ID: 731409). All patients were asked to give their written, informed consent prior to inclusion and data collection.

This study was carried out in two stages: First, the NPI was translated and cross‐culturally adapted into Norwegian. Second, the measurement properties of NPI‐No were examined in a cohort of patients 6 months after patellar stabilizing surgery.

### Translation and cross‐cultural adaption

The translation and cross‐cultural adaptation process followed internationally accepted guidelines described by Beaton et al. [3]. This process involved an expert committee of three orthopaedic surgeons, four physiotherapists specializing in orthopaedic rehabilitation, a researcher with extensive experience in clinimetric research methodology, a teacher specialized in the Norwegian language, and two back‐translators, both native speakers of English. The expert committee maintained close contact with the author of the original version throughout the process.

### Population

From January 2021 to October 2023, patients undergoing surgical treatment for recurrent patellar dislocation were recruited from two Norwegian Orthopaedic Centres. They were eligible for participation if they were ≥13 years old, fluent in Norwegian, and able to understand and complete the questionnaires. Patients with concomitant knee ligament injuries were excluded.

#### The NPI score

The NPI‐No score assesses activities of daily living as well as sports, encompassing movements of both high and low energy, including uniplanar and multi‐directional movements such as turning to look over the shoulder and twisting during sports. It comprises 19 questions assessed on a 5‐point Likert scale, ranging from ‘never’ to ‘always’. The sum score ranges from 0 to 250 and is presented as a percentage, where a higher score indicates higher instability [27, 29].

#### Concurrent measures used in hypotheses testing

The BPII 2.0 measures knee‐related quality‐of‐life (QoL). The questionnaire is specific and covers five domains: Symptoms and Physical Complaints, Work and/or School Related Concerns, Recreation/Sport/Activity, Lifestyle and Social and Emotional [15]. The International Knee Documentation Committee Subjective Knee Form (IKDC) 2000 is a knee‐specific, patient‐reported instrument, including three domains symptoms, physical activity and function [14]. The Knee Injury and Osteoarthritis Outcome Score (KOOS) is developed to evaluate patients' perspectives on their knee function and related problems. It consists of five subscales: pain, other symptoms, function in daily living, function in sports and recreational activities and knee‐related QoL [23]. The Tampa Scale of Kinesiophobia (TSK) was originally designed to assess fear of movement in patients with low back pain [17], but it has also been used to measure fear of re‐injuries in patients after medial patellofemoral ligament reconstruction [25].

The lower quarter Y‐balance test (YBT‐LQ) evaluates knee stability and one‐legged dynamic balance in three directions (anterior, posteromedial and posterolateral). Reach distance is normalized to leg length, which is measured from the anterior superior iliac spine to the most distal portion of the medial malleolus. Standing on one leg, patients reach as far as possible in each direction without losing their balance and mean reach distance of three attempts is recorded in centimetres (cm). Results are presented as a composite score of all three directions (in per cent) [12, 22, 24]. Single‐legged hop for distance evaluates function, dynamic strength and lower extremity muscle power. A single‐leg hop for distance is measured in cm, and results are presented as a mean of three trials [20]. Knee extension strength was measured with an isokinetic device (Biodex system 4 dynamometer, Biodex Medical Systems Inc.), using a standardized protocol of five repetitions at 60°/s. Results are presented as absolute values (in Newton metre, Nm).

#### Examination of measurement properties

Measurement properties of the NPI‐No were examined according to the guidelines of COnsensus‐based Standards for the selection of health Measurement INstruments (COSMIN) [18, 32].

*Face validity*, the degree to which the questionnaire seems to map the construct in a sufficient way [19] and *cultural adaptation* of the Norwegian version were assessed by the expert committee before the final version was used in the validation process.

*Content validity*, the degree to which the content of the NPI is an adequate reflection of the construct to be measured [19], was assessed by interviewing the first 10 patients as part of the pilot testing of the pre‐final version. ‘Think aloud’ interviewing [35] was applied when completing the NPI‐No before patients were asked about their interpretation of each item, relevance and any ambiguous wording and overall importance for their self‐perceived patellar instability.

C*onstruct validity* was assessed by testing predefined hypotheses about the expected direction and magnitude of correlations between scores on the NPI‐No and scores of instruments that measure similar constructs [18]. The predefined hypotheses were based on former validation studies on NPI [1, 10, 29, 33], and clinical experience (see Table 1). We expected IKDC 2000, to have a medium negative correlation to the NPI‐No, and the KOOS subscales to have a medium to large negative correlation to the NPI‐No. As patellar instability is assumed to affect QoL (BPII 2.0) and Kinesiophobia (TSK), we expected a medium to large negative correlation between BPII 2.0 and NPI‐No, and a small to medium correlation to the TSK. We expected medium negative correlation between physical tests and NPI‐No because physical tests and self‐reported function measure similar but distinct aspects of physical function.

**Table 1:** Hypotheses about expected associations between the NPI and measures of knee function.

There is a medium to large negative correlation (−0.30 < *r* < −1.0) between BPII 2.0 and NPI
There is a medium negative correlation (−0.30 < *r* <−0.50) between IKDC 2000 and NPI
There is a small to medium correlation (0.1 < *r* < 0.5) between TSK and NPI
There is a medium to large negative correlation (−0.30 < *r* < −1.0) between KOOS QoL and NPI
There is a medium to large negative correlation (−0.30 < *r* < −1.0) between KOOS Pain and NPI 0
There is a medium to large negative correlation (−0.30 < *r* < −1.0) between Sport/Rec and NPI
There is a medium to large negative correlation (−0.30 < *r* < −1.0) between KOOS Symptoms and NPI
There is a medium to large negative correlation (−0.30 < *r* < −1.0) between KOOS ADL and NPI
There is a medium negative correlation (−0.30 < *r* <−0.50) between functional tests and NPI
There is a medium negative correlation (−0.30 < *r* <−0.50) between quadriceps strength and NPI

Abbreviations: BPII, Banff Patellofemoral Instability Instrument; IKDC 2000 Knee Documentation Committee Subjective Knee Form 2000; KOOS The Knee injury and Osteoarthritis Outcome Score; NPI, Norwich Patellar Instability; QoL, quality‐of‐life; TSK Tampa Scale of Kinesiophobia.

*Internal consistency*, defined as ‘the degree of interrelatedness among the items’ [19], was assessed by Cronbach's alpha coefficient (*α*); 0.70 is acceptable, ≥0.80 is preferable and >0.95 might indicate item redundancy [32].

A *floor or ceiling effect* is considered to be present if the amount of minimal or maximal scores exceeds 15% [7]. The NPI was examined for floor and ceiling effects for the scale as a whole both before surgery and 6 months post‐operatively.

Test–retest reliability, the reproducibility of the questionnaire, was examined in a subgroup of 60 patients who completed the NPI‐No 2 weeks prior to—and at the time of—the 6‐month post‐operative follow‐up.

### Data processing and statistical analyses

SPSS 26.0 (IBM Corp.) was used for data analyses. These included descriptive statistics, testing of normality, examination of internal consistency, test–retest reliability, Bland–Altman plots, hypothesis testing (significance level *p* ≤ 0.05) and floor and ceiling effects. For continuous variables, means and standard deviations (SDs) are presented and for categorical variables absolute and relative frequencies are presented. Measurement errors were calculated in Microsoft Excel (Microsoft, 2016).

Reliability was calculated using intraclass correlation coefficient (ICC_2.1_) with 95% confidence intervals (CIs) based on two‐way random, single measures with absolute agreement [32]. An ICC of 0.70–0.89 indicate high correlation and 0.90–1.00 indicate very high correlation [7, p. 120]. *Standard error of measurement (SEM)*, was calculated from the mean of the variance between tests [32]. A 95% CI of SEM was made to suggest the limits of measurement error (1.96*SEM). To express the smallest change score (with *p* ≤ 0.05) that can be interpreted as a real change and not measurement error, smallest detectable change (SDC) at individual level (SDC_ind_) was calculated based on SEM (1.96 × √2 × SEM). The SDC on group level (SDC_group_) was calculated as (SDC_ind_/√*n*) [32]. To evaluate limits of agreement (LoA), a Bland–Altman plot was used [7, p. 113]. Correlations were investigated using Pearson's *r*; 0.10–0.29 were considered small, 0.30–0.49 medium and 0.50–1.0 large [5, pp. 79–81]. Sample size determination was based on recommendations from Terwee et al. [32]— suggesting a minimum of 50 patients for assessing construct validity, reliability and floor or ceiling effects, and a minimum of 100 patients for assessing internal consistency [7, p. 191].

## RESULTS

**Figure 1:**
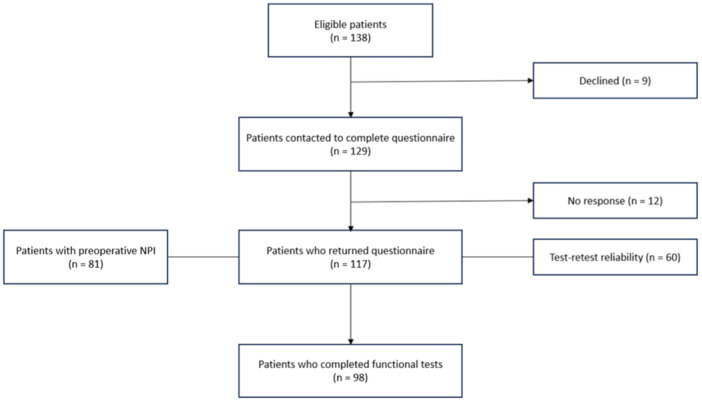
Flowchart of patient's participation.

One hundred seventeen patients met the inclusion criteria and volunteered for the study (see Figure 1 for flowchart). All patients completed the battery of questionnaires 6 months post‐operatively; of these, 81 completed the NPI score before surgery, and a subgroup of 98 patients underwent functional testing at 6 months after surgery. The study population comprised 71% females and mean age was 22.8 ± 7.3 years at surgery. Fifty‐three per cent of the patients had bilateral instability and mean time from the first dislocation episode (to the time of surgery) was 7.2 years (range 0–27 years). Patient characteristics are presented in Table 2.

**Table 2:** Patient characteristics at time of surgery (*N* = 117).^a^

Age at surgery, years	22.8 ± 7.3
Female sex	83 (71)
Years since first dislocation	7.2 ± 6.3
Bilateral problems	62 (53)
Surgical procedure	
Isolated MPFL‐R	21 (17.9)
Combined surgery	96 (82.1)

Abbreviations: BMI, body mass index; MPFL‐R, medial patellofemoral ligament reconstruction; SD, standard deviation.

^a^ Data are reported as *n* (%) or mean ± SD unless otherwise noted.

### Data quality

All patients were able to complete the NPI‐No without assistance, and only one patient had an item missing. The mean NPI score was 34.1 (SD = 19.0, range = 1.6–100) before surgery and 11.1 (SD = 17.2, range = 0–88) 6 months after surgery, displaying that patients reported significantly less patellar instability 6 months after surgery. There was no ceiling effect pre‐ or post‐operatively, as only *one* patient had the worst possible score (100) preoperatively. While no floor effect was seen before surgery, 28% had the best possible score (0) at the 6‐month follow‐up, meaning that a substantial floor effect was present.

### Measurement properties

The expert committee agreed that the NPI‐No had sufficient face validity. However, there were some concerns about the formula for the calculation of the sum score. This was related to questions related to challenging activities where patients could answer ‘do not’ and still get a sum score of zero if their knee felt stable in other, less demanding activities. Further, support for good c*ontent validity* was found as interviewed patients reported: (1) comprehensible instructions and questions, (2) a high relevance of the included items and (3) no missing key aspects of their patellar instability.

The NPI‐No displayed medium negative correlation to the BPII 2.0 (*r* = −0.432), the IKDC (*r* = −0.367) and four out of five KOOS subscales (*r* = −0.330 to −0.385). The KOOS subscale Sport/Rec only demonstrated a small negative association with the NPI‐No (*r* = −0.292). Further, a small positive correlation with the TSK (*r* = 0.149) was evident. There was no correlation between physical tests (YBT‐LQ, hop tests and strength tests) and the NPI‐No. In total, 7 of the 10 pre‐defined hypotheses were confirmed, supporting fairly good *construct validity* (Table 3).

**Table 3:** Descriptive statistics on measurements used in hypothesis testing at 6‐month follow‐up (*N* = 116).^a^

			Correlation analysis	
Hypothesis	Measurements	Mean (SD), min–max	Pearson's *r*	*p*
1	BPII 2.0^b^	63.9 (20.2), 18.8–98.2	−0.432	0.001
2	IKDC 2000^c^	67.4 (15.6), 33.3–100	−0.367	0.001
3	TSK^c^	26.9 (6.6), 13.0–44.0	0.149	0.05
4	KOOS Pain^c^	81.7 (15.6), 38.0–100	−0.330	0.001
5	KOOS Symptoms^c^	76.1 (14.4), 31.0–100	−0.363	0.001
6	KOOS ADL^c^	91.5 (10.2), 60.0–100	−0.385	0.001
7	KOOS Sport/Rec^c^	60.5 (25.5), 5.0–100	−0.292	0.001
8	KOOS QoL^c^	57.4 (21.2), 6.0–100	−0.367	0.001
9	YBT‐LQ Composite reach operated leg %^d^	75.9 (9.7), 52.7–100	−0.063	n.s
9	Singel‐legged hop test for distance operated leg, cm^f^	72.0 (35.8), −1.5 to 145.0	−0.070	n.s
10	PT extension 60°/s operated leg, Nm^e^	94.5 (41.7), 13.0–198.6	−0.179	n.s

Abbreviations: BPII, Banff Patellofemoral Instability Instrument; cm, centimetre; IKDC 2000, Knee Documentation Committee Subjective Knee Form 2000; KOOS, The Knee Injury and Osteoarthritis Outcome Score; Nm, Newton metre; NPI, Norwich Patellar Instability; PT, peak torque; QoL, quality‐of‐life; TSK, Tampa Scale of Kinesiophobia.

^a^ One outlier is removed from the analysis.

^b^ *N* = 115.

^c^ *N* = 112.

^d^ *N* = 96.

^e^ N = 94.

^f^ *N* = 89.

**Figure 2:**
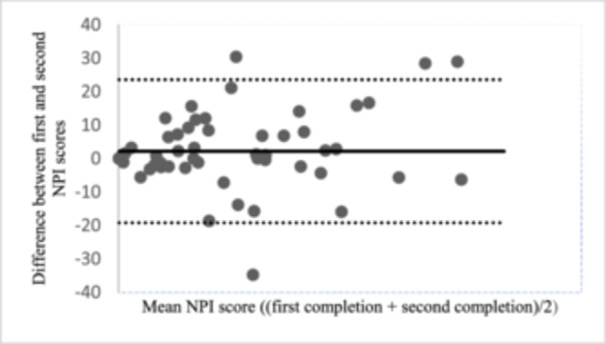
Bland–Altman plot displaying limits of agreement (*n* = 60). NPI–No, Norwich Patellar Instability score Norwegian version.

*Internal consistency* was satisfactory for the total NPI score, with Cronbach's *α* of 0.88 (*n* = 116). The α value varied from 0.87 to 0.88 if any of the items were deleted, indication no redundancy. *Test re‐test reliability* of the NPI‐No was moderate, with an ICC_2.1_ value of 0.65 (0.47–0.77) (Table 4). SEM was 7.8 implicating that a change in score for one individual must exceed 21.7 points (SDC_ind_) and on group level 2.8 (SDC_group_) points to be interpreted as a true change (exceeding measurement error). A graphic presentation of LoA is presented in a Bland–Altman plot (Figure 2), upper limit was 23.6 and lower limit was −19.2 points.

**Table 4:** Test re‐test reliability, measurement error and SDC of the NPI‐No (*N* = 60).

NPI‐No. 1 administration, mean (SD)	12.0 (12.0)
NPI‐No. 2 administration, mean (SD)	14.1 (14.1)
Mean difference	1.9
ICC 2.1. (95% CI)	0.65 (0.47–0.77)
SEM	7.81
1.96*SEM	15.32
SDC individual	21.66
SDC group	2.80

Abbreviations: CI, confidence interval; ICC, intraclass correlation coefficient; NPI, Norwich Patellar Instability; SD, standard deviation; SDC, smallest detectable change; SEM, standard error of measurement.

## DISCUSSION

The NPI was successfully translated and cross‐culturally adapted to Norwegian‐speaking patients with patellar instability. The current study indicates that the NPI‐No is relevant and comprehensible, with adequate construct validity, sufficient internal consistency and moderate reliability. An SDC of 21.7 points means that score changes in one individual need to exceed this number to be interpreted as a ‘true’ change.

No floor or ceiling effects were observed for the NPI before surgery. However, a substantial floor effect was found in the post‐operative results, as 28% reached the best possible score. Considering that the goal of patella stabilizing surgery is to provide patients with a stable patella, one would expect a high number of patients achieving the best possible score post‐operatively. Interestingly, no floor effect was found in a recent study by Yilmaz et al. [36] and only a 13% floor effect was found at 12 months post‐operatively in a study from the original developer [28]. The reason for this discrepancy is unknown, however, based on the current finding it is reasonable to question of whether a score of zero truly reflects the best possible outcome and if it is meaningful to measure beyond this point especially in higher functioning individuals.

Support for fairly good construct validity was found as seven out of ten hypotheses were confirmed. The NPI showed, as presumed, a moderate negative association with the BPII 2.0 (*r* = −0.432). This finding is comparable to other studies examining concurrent validity between the NPI and the BPII 2.0 [4, 10, 13]. Further, self‐perceived knee function (IKDC) was moderately negatively associated with NPI (*r* = −0.367), reflecting that, even though patellar instability affects many activities of daily life and sports, it comprises only one aspect of patients' self‐perceived knee function. As the NPI was derived from activities that patients with patellar instability reported would make their patella unstable, the small association between kinesophobia measured by the TSK and the NPI (*r* = 0.149) was somewhat surprising. This means that patients with a stable patella can have a high degree of kinesophobia and vice versa. It further implies that the two scores are assessing distinct constructs with minor overlap.

The moderate to small associations of NPI to all KOOS subscales are interesting since the subscales ADL and Sport/Rec have several similar items as the NPI, such as questions about getting into or out of a car, walking on even surfaces, hopping, and jumping, and therefore a higher association was expected. The reason might be due to the fact that KOOS was originally designed to assess knee osteoarthritis and its associated functional limitation [23]. It is also noteworthy that previous studies examining the associations between the KOOS and the NPI have yielded divergent results. While the original version of the NPI demonstrated only small associations with the KOOS subscales [29], the Dutch version showed significant associations with all subscales [33]. If this discrepancy is due to different cultures, populations (gender, age and level of activity) or methodology, for example, timing is not known.

Since this is the first study that also investigates associations between physical tests and NPI, there were no studies to inform hypothesis formation about what associations to expect. Nonetheless, it was assumed that the degree of self‐perceived instability would have a moderate to large negative association with performance on the different tests because the tests are designed to challenge dynamic stability. This was not supported in the current study, as no association between any of the physical tests and the NPI‐No was found. A possible explanation might be that patellar instability is not sufficiently challenged in the included physical tests or that there is a mismatch between the patient's perceived instability and actual performance. In the former case, the fact that these patients were tested post‐operatively might play a role. If testing had been done in a population with residual instability that had not undergone stabilizing surgery, the relation between physical tests and the self‐perceived instability might have been different.

The internal consistency of the NPI was satisfactory (*α* = 0.88) and in line with former studies [29, 36]. This indicates that the 19 items are interrelated and consistently measure the same construct. Some of the inter‐item correlations were above the limit of 0.7 (Supporting Information S1: Appendix 1), described by de Vet et al. [7], indicating that these items measure almost the same thing, that is, capture the same aspects of self‐perceived patellar instability, and therefore are redundant.

Only one other study has presented results on test–retest reliability of the NPI [36]. The 2‐week interval between completions should be long enough to prevent recall and short enough to minimize the risk of changes in the patient's condition, meaning that we expected their self‐perceived instability to be stable in this relatively short time span. The moderate ICC is below the minimum acceptable value of 0.70 recommended by de Vet et al. [7, p. 120], indicating that the reproducibility of the NPI score might only be moderate and interpretation of changes should therefore be done with care. There is, however, one study reporting excellent ICC values [36]. One could only speculate whether this discrepancy is because the response categories are too vague and therefore patients interpret them different in each completion or if measurement overload in the current study are contributing factor.

Another factor to consider when evaluating the responsibility of a scale is absolute reliability or measurement error as expressed by SEM and SDCind. These have formerly not been reported on for the NPI score. The estimated SDC_ind_ in the current study indicates that only a change higher than 21.66 points on the NPI can be considered a “real” change for the individual patients and be distinguished from measurement error. This study is also the first to report the 95% LoA for the NPI, which visually illustrates the magnitude of measurement error. Interpretation of score changes on the NPI is, however, still challenging due to the lack of an established minimal important change (MIC), consequently, future studies should address this limitation and consider establishing MIC for NPI score.

One strength of this study is the prospective inclusion of patients. The relatively high standard deviations observed in the analysis are consistent with previous research [27, 28, 33, 36] and suggest heterogeneous population which is typical for patients with patellar instability patients [2, 27, 34]. Furthermore, we believe that the inclusion of 85% of all patients undergoing surgical treatment for recurrent patellar dislocation in two orthopaedic units provides a representative and unselected, cohort of this group of patients. This is a strength as it gives a good generalizability of the current results. Other strengths are a sufficient number of patients according to current recommendations and a predefined hypothesis to test construct validity, including a hypothesis that has not been evaluated in former studies. Also, in the process of evaluating the effect of surgery where the goal is to provide a stable patella, mapping self‐perceived patellar instability is a reasonable tool to use.

There are some limitations to consider. This study was conducted in only two orthopaedic units; a multicentre study would increase the scientific value and provide more diverse perspective. Comparison to other cross‐cultural validation studies of the NPI is limited due to the limited number of published studies available (only two) [33, 36]. This lack of comparative data affects the interpretation of results and highlights the need for research in this area. While the IKDC has been validated in patients with patellar instability, demonstrating satisfactory reliability, the presence of a notable ceiling effect may compromise its validity [21]. Further, the existing Norwegian versions of the IKDC and KOOS utilized in this study lack the recommended assessment of measurement properties [9, 11], which raises concerns about its reliability and validity. The TSK is assumably measuring relevant aspects for patients with patellar instability (fear of movement). However, there is limited information on the validity and reliability of the TSK scores in patients with patellar instability, which warrants caution in interpretation. Overall, these questionnaires are in extensive use and widely accepted in both research and clinical settings despite the limited information on the measurement properties of the Norwegian versions. The functional tests are also widely employed after knee injuries; however, their validity and reliability in patients with patellar instability are not yet established. Therefore, it is crucial to acknowledge the limitations in the comparative use of these instruments.

## CONCLUSION

The translation and cross‐cultural adaptation of the NPI score into Norwegian was successfully accomplished in this study. The support for content validity indicates that the items of the NPI‐No reflect relevant and important aspects of self‐perceived patellar instability. The NPI‐No also demonstrates fairly good construct validity and sufficient internal consistency, indicating that it measures the intended construct well and that the items are consistently related to each other. However, the reproducibility was found to be only moderate in this study. These findings contribute to the growing body of evidence on the validity and reliability of NPI scores in accordance with the COSMIN guidelines.

## AUTHOR CONTRIBUTIONS

**T. Hysing‐Dahl:** Conception and design of study, acquisition of data, analysis and interpretation of data and drafting of manuscript. **A. G. Faleide:** Conception of study, acquisition, analysis and interpretation of data and critically revising the manuscript. **L. H. Magnussen:** Conception of study, acquisition, analysis and interpretation of data and critically revising manuscript. **E. Inderhaug:** Conception and design of study, acquisition of data, analysis and interpretation of data and critically revising manuscript.

## CONFLICT OF INTEREST STATEMENT

The authors declare no conflict of interest.

## ETHICS STATEMENT

The study was approved by the NSD (Norwegian Centre for Research Data) Data Protection Official for Research, project number 731409 and the Regional Committee for Medical and Health Research Ethics (ID: 2020/185067). Informed consent: yes.

## Supporting information

Supplementary Information

## Data Availability

Data are available on reasonable request. Quotations and further details are available from TH‐D at Trine. Hysing-Dahl@haraldsplass.no.
