# Supplementary material for: The Norwegian version of the Norwich Patellar Instability score has good validity and moderate reproducibility
Source: J Exp Orthop. 2025 Jan 10;12(1):e70095. doi: 10.1002/jeo2.70095 (PMC11718546; doi:10.1002/jeo2.70095)
Supplement: Supplementary file 1 — Supplementary Information [file JEO2-12-e70095-s001.pdf]

Appendix 1

Inter-Item Correlation Matrix

	Q1	Q2	Q3	Q4	Q5	Q6	Q7	Q8	Q9	Q10	Q11	Q12	Q13	Q14	Q15	Q16	Q17	Q18	Q19
Q1	1.000	.449	.279	.539	.290	.098	.166	.300	.265	.127	.181	.276	.311	.210	.011	.109	.190	.286	.312
Q2	.449	1.000	**.757**	.275	**.716**	.596	.544	**.794**	-.119	.419	.289	-.007	-.006	.184	.165	-.008	.001	.047	.111
Q3	.279	.757	1.000	.324	.666	.563	.509	**.745**	-.090	.325	.251	.020	.005	.098	.013	-.010	-.076	-.011	.067
Q4	.539	.275	.324	1.000	.234	.257	.244	.334	.416	.260	.319	.458	.417	.379	.203	.332	.302	.322	.275
Q5	.290	.716	.666	.234	1.000	.711	.606	.634	-.032	.320	.330	.060	.079	.213	.122	.003	.087	.142	.106
Q6	.098	.596	.563	.257	.711	1.000	**.731**	.573	-.096	.432	.419	.015	-.021	.239	.132	-.001	-.018	.105	.038
Q7	.166	.544	.509	.244	.606	**.731**	1.000	.473	.008	.432	.457	.088	-.044	.298	.102	.073	-.080	-.073	.027
Q8	.300	**.794**	**.745**	.334	.634	.573	.473	1.000	-.113	.303	.224	.053	-.015	.000	.060	.032	.026	.046	.112
Q9	.265	-.119	-.090	.416	-.032	-.096	.008	-.113	1.000	.222	.215	**.771**	.793	.464	.356	.617	.715	.549	.485
Q10	.127	.419	.325	.260	.320	.432	.432	.303	.222	1.000	.651	.339	.215	.540	.426	.302	.217	.270	.274
Q11	.181	.289	.251	.319	.330	.419	.457	.224	.215	.651	1.000	.227	.184	.478	.371	.234	.175	.304	.271
Q12	.276	-.007	.020	.458	.060	.015	.088	.053	**.771**	.339	.227	1.000	**.723**	.411	.364	**.718**	.656	.463	.515
Q13	.311	-.006	.005	.417	.079	-.021	-.044	-.015	**.793**	.215	.184	**.723**	1.000	.409	.384	.605	.715	.607	.538
Q14	.210	.184	.098	.379	.213	.239	.298	.000	.464	.540	.478	.411	.409	1.000	.505	.424	.370	.376	.416
Q15	.011	.165	.013	.203	.122	.132	.102	.060	.356	.426	.371	.364	.384	.505	1.000	.469	.409	.388	.448
Q16	.109	-.008	-.010	.332	.003	-.001	.073	.032	.617	.302	.234	**.718**	.605	.424	.469	1.000	.663	.462	.518
Q17	.190	.001	-.076	.302	.087	-.018	-.080	.026	**.715**	.217	.175	.656	**.715**	.370	.409	.663	1.000	.651	.608
Q18	.286	.047	-.011	.322	.142	.105	-.073	.046	.549	.270	.304	.463	.607	.376	.388	.462	.651	1.000	.590
Q19	.312	.111	.067	.275	.106	.038	.027	.112	.485	.274	.271	.515	.538	.416	.448	.518	.608	.590	1.000

Bold values are > 0.70

Følsomhet Intern (gul)
